# The oncolytic virus *dl*922-947 reduces IL-8/CXCL8 and MCP-1/CCL2 expression and impairs angiogenesis and macrophage infiltration in anaplastic thyroid carcinoma

**DOI:** 10.18632/oncotarget.6430

**Published:** 2015-11-29

**Authors:** Carmela Passaro, Francesco Borriello, Viviana Vastolo, Sarah Di Somma, Eloise Scamardella, Vincenzo Gigantino, Renato Franco, Gianni Marone, Giuseppe Portella

**Affiliations:** ^1^ Department of Translational Medical Sciences, University of Naples Federico II, Naples, Italy; ^2^ Center for Basic and Clinical Immunology Research (CISI), University of Naples Federico II, Naples, Italy; ^3^ CNR Institute of Experimental Endocrinology and Oncology “G. Salvatore”, Naples, Italy; ^4^ Experimental Oncology, IRCCS Fondazione Pascale, Naples, Italy

**Keywords:** virotherapy, tumor vasculature, innate immunity, tumor-associated macrophages, chemokine

## Abstract

Anaplastic thyroid carcinoma (ATC) is one of the most aggressive human solid tumor and current treatments are ineffective in increasing patients' survival. Thus, the development of new therapeutic approaches for ATC is needed. We have previously shown that the oncolytic adenovirus *dl*922-947 induces ATC cell death *in vitro* and tumor regression *in vivo*. However, the impact of *dl*922-947 on the pro-tumorigenic ATC microenvironment is still unknown. Since viruses are able to regulate cytokine and chemokine production from infected cells, we sought to investigate whether *dl*922-947 virotherapy has such effect on ATC cells, thereby modulating ATC microenvironment. *dl*922-947 decreased IL-8/CXCL8 and MCP-1/CCL2 production by the ATC cell lines 8505-c and BHT101-5. These results correlated with *dl*922-947-mediated reduction of NF-κB p65 binding to *IL8* promoter in 8505-c and BHT101-5 cells and *CCL2* promoter in 8505-c cells. IL-8 stimulates cancer cell proliferation, survival and invasion, and also angiogenesis. *dl*922-947-mediated reduction of IL-8 impaired ATC cell motility *in vitro* and ATC-induced angiogenesis *in vitro* and *in vivo*. We also show that *dl*922-947-mediated reduction of the monocyte-attracting chemokine CCL2 decreased monocyte chemotaxis *in vitro* and tumor macrophage density *in vivo*. Interestingly, *dl*922-947 treatment induced the switch of tumor macrophages toward a pro-inflammatory M1 phenotype, likely by increasing the expression of the pro-inflammatory cytokine interferon-γ. Altogether, we demonstrate that *dl*922-947 treatment re-shape the pro-tumorigenic ATC microenvironment by modulating cancer-cell intrinsic factors and the immune response. An in-depth knowledge of *dl*922-947-mediated effects on ATC microenvironment may help to refine ATC virotherapy in the context of cancer immunotherapy.

## INTRODUCTION

Tumorigenesis is a multistep process that involves cancer cell-intrinsic alterations and complex interactions with tumor stroma and infiltrating immune cells [1, 2]. Although therapeutic strategies aimed to target specific features of this process have proved to be effective for cancer treatment, their curative potential is often hampered by the development of a variety of resistance mechanisms. These involve acquisition of alternative strategies to restore the feature targeted by the treatment or reliance on other mechanisms. Indeed, cancer cell-extrinsic factors provided by the tumor microenvironment may mediate resistance to cytotoxic therapies. Thus, it is conceivable that strategies aimed at co-targeting several tumor-promoting mechanisms (e.g. cancer cell-intrinsic features and the tumor microenvironment) could optimize effectiveness and reduce therapeutic escape [3].

Oncolytic viruses (OVs) are non-pathogenic viral strains or viral mutants that selectively replicate in and kill tumor cells without causing harm to normal cells [4]. The efficacy and safety of OVs has been demonstrated in clinical studies with encouraging results [5]. Recently, The FDA has approved the first oncolytic viral therapy, Talimogene laherparepvec (T-VEC), to treat surgically unresectable skin and lymph node lesions in patients with advanced melanoma [6].

The mechanism of action of OVs has long been thought to be solely dependent on direct tumor cell killing. A growing body of evidence points to the immune system as having a critical role in determining the efficacy of virotherapy [7-9]. Indeed, OVs may break the tolerogenic tumor microenvironment and induce a long-lasting CD8 T cell-mediated anti-tumor response, thereby acting as vaccines [7]. In certain cases, a robust immune-dependent anti-tumor response was also elicited independently of viral oncolysis and replication [10]. Based on this evidence, several viral strains have been engineered to express factors (e.g. cytokines, chemokines and membrane receptors) that boost the anti-tumor immune response of which several have already shown promising results in clinical trials [11-16]. Taken together, evidence obtained so far suggests that a complex interaction between OV replication and the response of both tumor and immune cells to OV infection eventually determines the outcome of virotherapy.

Anaplastic thyroid carcinoma (ATC) is one of the deadliest human solid tumors: although it accounts for less than 3% of all thyroid cancers, ATC is responsible for up to 50% of the annual mortality associated with thyroid tumors [17]. ATC treatment consists of surgery combined with radiation and chemotherapy. However, this treatment is often palliative as death usually occurs within 6 months from diagnosis, commonly caused by tracheal obstruction [18]. Several novel approaches have been tested for the treatment of ATC, including gene therapy [19, 20] and oncolytic virotherapy [21]. Our group has extensively evaluated the oncolytic adenovirus *dl*922-947 for the treatment of ATC. *dl*922-947 is a second generation adenoviral mutant bearing a 24-bp deletion in E1A-Conserved Region 2 [21]. This region binds to and inactivates pRb, dissociating the pRb-E2F complex and driving S phase entry and viral replication. The mutant E1A protein encoded by *dl*922-947 is unable to bind pRb, thereby the virus cannot induce S phase entry in normal cells. Nonetheless, *dl*922-947 replicates in cells with an aberrant G1-S checkpoint, which is a feature of almost 90% of human cancers [22]. In several experimental neoplastic models, including ATC, *dl*922-947 treatment, alone or in combination with molecularly-targeted drugs or ionizing radiations, can induce cancer cell death and tumor regression [23-30]. Although these studies were aimed to demonstrate synergistic effects of the combined treatments, we also observed an anti-angiogenic effect of the virus [29]. It is still unclear whether and how *dl*922-947 virotherapy modulates ATC microenvironment. Understanding the modulation of ATC microenvironment by *dl*922-947 treatment and the contribution to the anti-tumoral activity will be instrumental to develop more effective therapies.

ATC is markedly infiltrated by immune cells, namely macrophages [31, 32], which are likely to play a role in ATC development and progression. Tumor-associated macrophages (TAMs) may differentiate from peripheral blood monocytes recruited in a MCP-1/CCL2-dependent manner (hereafter CCL2) [33]. ATC cells express high levels of the enzyme indoleamine 2,3-dioxygenase 1 (IDO1) that catalyzes the conversion of amino acid tryptophan to the immunosuppressive molecule kynureine [34]. In addition, ATC cells produce the chemokine IL-8/CXCL8 (hereafter IL-8) that is involved in several aspects of tumor biology, namely cell proliferation, cell survival, epithelial-to-mesenchymal transition, stemness, and also angiogenesis [35-38].

Virus-infected cells activate an antiviral response aimed at blocking viral replication. The cellular antiviral response involves the production of type I and type III interferons (IFNs) and the modulation of cytokine and chemokine production. On the other hand, viruses have evolved several mechanisms to counteract the cellular antiviral mechanisms, e.g. by modulating the activity of transcription factors involved in the production of cytokine and chemokines [39, 40]. We hypothesized that *dl*922-947 virotherapy of ATC cells modulate the production of cytokines and chemokines, leading to modification of the tumor microenvironment.

In the present study we show that *dl*922-947 treatment reduces IL-8 and CCL2 production by ATC cell lines via displacement of the transcription factor NF-κB p65 from *IL8* and *CCL2* promoters. Furthermore, we provide evidence that *dl*922-947-induced reduction of IL-8 and CCL2 production correlates with impaired tumor angiogenesis and decreased macrophage density *in vitro* and *in vivo*.

## RESULTS

### *dl*922-947 reduces IL-8 and CCL2 production by ATC cell lines

**Figure 1:**
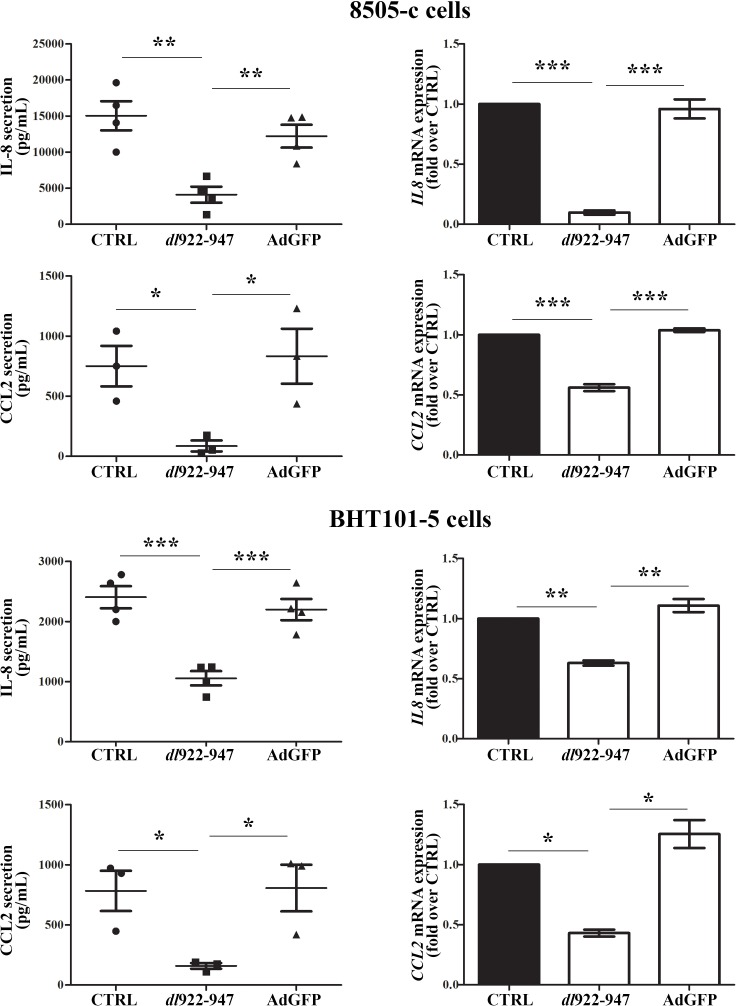
IL-8 and CCL2 secretion and expression upon *dl*922-947 treatment 8505-c and BHT101-5 cells were treated with *dl*922-947 or the non-replicating adenovirus AdGFP (5 an 1 pfu/cell for both viruses, respectively). 48 hours after infection IL-8 and CCL2 secretion (left panels) and expression (right panels) were assessed by ELISA on cell-free supernatants and Real-Time PCR, respectively. The results are the mean of three independent experiments ±SEM. One-way ANOVA and Tuckey post-test: *p<0.05; **p<0.01; ***p<0.001.

The cellular response to viral infections involves the production of IFNs and the modulation of cytokines and chemokines release. Therefore, we analyzed by ELISA whether *dl*922-947 treatment of the ATC cell lines 8505-c and BHT101-5 modulates the expression of cytokines, chemokines and angiogenic factors. We could not detect TNF or IL-10 secretion in any of the tested conditions (data not shown), while VEGF-A production was not significantly modified by *dl*922-947 compared to the replication-defective adenovirus AdGFP (Supplementary Figure 1). Interestingly, both cell lines produced high levels of IL-8 and CCL2, which were reduced upon treatment with *dl*922-947 but not AdGFP (Figure 1, left panels). The latter results were confirmed by Real-Time PCR analysis of mRNA levels (Figure 1, right panels). In addition, we observed the same pattern of IL-8 and CCL2 secretion when the concentrations of these chemokines were normalized on the total cellular protein content to account for *dl*922-947-induced cell death (Supplementary Figure 2A).

In several cell types, the constitutive- or stimuli (e.g. PMA or TNF)-induced expression of IL-8 is negatively regulated by IFNβ at the transcriptional level [41-43]. Furthermore, virus-infected cells produce IFNβ as part of their antiviral response program [40]. Thus, we investigated whether IFNβ was involved in the *dl*922-947-dependent reduction of IL-8 production by acting in an autocrine/paracrine manner. IFNβ treatment did not reduce IL-8 production (Supplementary Figure 2B), nor IFNβ secretion could be detected by *dl*922-947-infected ATC cell lines (data not shown).

### IL-8 and CCL2 reduction is associated with NF-κB p65 displacement from *IL8* and *CCL2* promoters

**Figure 2:**
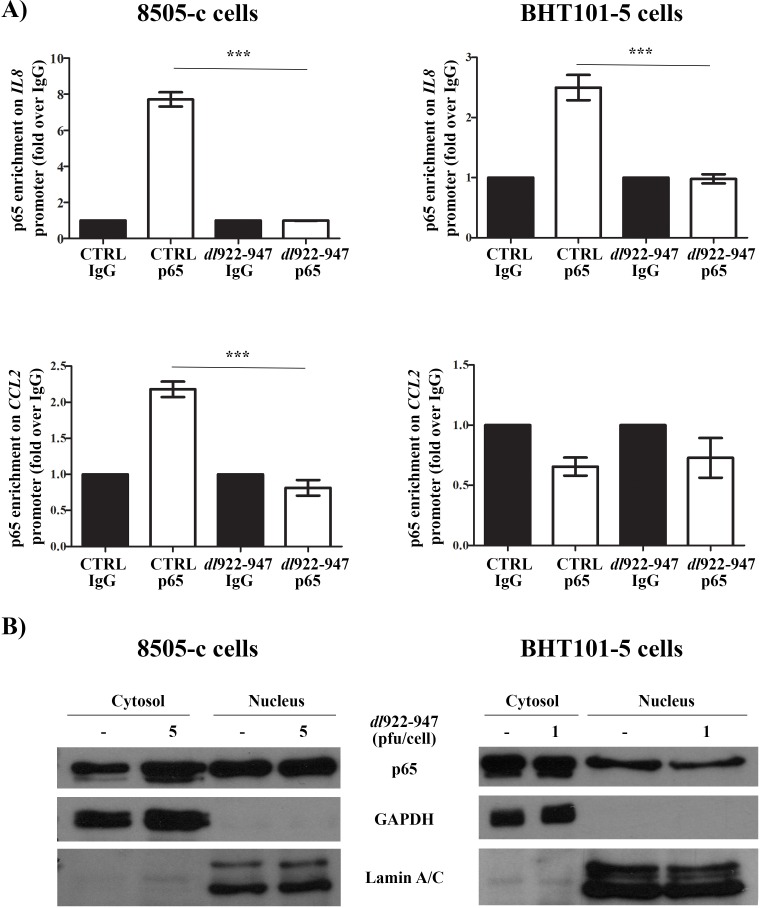
p65 localization and binding to *IL8* and *CCL2* promoters after *dl*922-947 treatment **A**. 8505-c and BHT101-5 cells were treated with *dl*922-947 (5 or 1 pfu/mL, respectively). After 24 hours, p65 binding to *IL8* (upper panels) and *CCL2* (lower panels) promoters was assessed by chromatin immunoprecipitation (ChIP) assay. For each experimental condition, p65 binding is expressed as fold enrichment over the non-specific binding (IgG) control. The results are the mean of three independent experiments ±SEM. One-way ANOVA and Tuckey post-test: ***p<0.001. **B**. 8505-c and BHT101-5 cells were treated for 24 hours with *dl*922-947 (5 and 1 pfu/cell, respectively). Cellular fractionation was performed to isolate nuclear and cytosolic fraction. Lysates were probed with p65 antibody, as well as Lamin A/C and GAPDH antibodies to verify the purity of the preparations. The blots are representative of three independent experiments.

The expression of *IL8* and *CCL2* genes is regulated by NF-κB p65 (hereafter p65) nuclear localization and binding to *cis*-regulatory elements in *IL8* and *CCL2* promoters [44-46]. *dl*922-947 reduced p65 binding to *IL8* (in BHT101-5 and 8505-c cells) and *CCL2* (in 8505-c) promoters, as assessed by chromatin immunoprecipitation (ChIP) assay (Figure 2A), without affecting total p65 protein levels (Supplementary Figure 3A). Cellular fractionation showed that *dl*922-947 treatment reduced p65 nuclear localization in BHT101-5 (Figure 2B). This effect was not observed in 8505-c cells (Figure 2B), indicating an alternative mechanism of p65 modulation.

**Figure 3:**
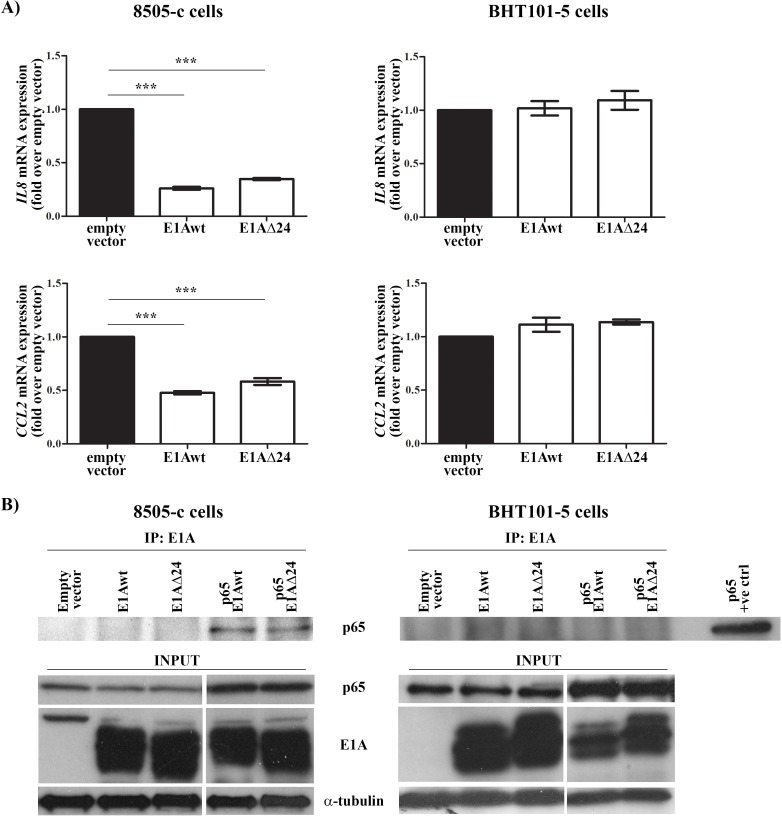
E1A-dependent and -independent modulation of *IL8* and *CCL2* gene expression **A**. 8505-c and BHT101-5 cells were transfected with wild type (E1Awt) or a mutated form (E1AΔ24) of the adenoviral protein E1A. 32 hours after transfection *IL8* and *CCL2* mRNA expression was evaluated by Real-Time PCR. The results are the mean of three independent experiments ±SEM. One-way ANOVA and Tuckey post-test: ***p<0.001. **B.** 8505-c and BHT101-5 cells were transfected with E1Awt or E1AΔ24 alone or co-transfected with p65. 32 hours after transfection E1A was immunoprecipitated and its binding to p65 was assessed by western blotting using an anti-p65 antibody. The levels of p65, E1A and α-tubulin in total cell lysates were included as input controls. A BHT101-5 cells only transfected with p65 was used as positive control (p65 +ve control). The blots are representative of three independent experiments.

Adenoviral proteins (e.g. E1A, E1B19K, E3) are known to interact with p65, impairing its activity [47-49]. In addition, p65 binds to *E3* promoter and induces the expression of E3 proteins [50]. *dl*922-947 is completely deleted in *E310.4K, E314.5K and E314.7K* genes, whereas it has only a 24 bp deletion in the pRb-interacting region of *E1A* gene (E1AΔ24) [51]. To assess which adenoviral protein mediates the effects of *dl*922-947, we treated both cell lines with the E1B19K-deleted adenovirus Δ19K that express an intact form of E1A. The mutant Δ19K reduced *IL8* mRNA expression (Supplementary Figure 3B), excluding E1B19K involvement in p65 modulation. Then, ATC cell lines were transfected with plasmids expressing E1A wild type (E1Awt) or a CR2-deleted form of E1A (E1AΔ24). The efficiency of transfection was confirmed by assessing *E1A* mRNA levels in transfected cells (data not shown). Transfection of E1Awt and E1AΔ24 reduced *IL8* expression in 8505-c but not in BHT101-5 cells (Figure 3A). To evaluate a direct interaction between E1A and p65, protein extracts were immunoprecipitated using an anti-E1A antibody and the binding to p65 was assessed using an anti-p65 antibody. The interaction of E1Awt and E1AΔ24 with p65 was only observed in 8505-c cells (Figure 3B). Taken together, our data indicate that *dl*922-947 reduces p65 binding to *IL8* promoter through an E1A-dependent mechanism in 8505-c cells or E1A-independent mechanisms that leads to reduced p65 nuclear localization in BHT101-5 cells.

We also evaluated p65 binding to *CCL2* promoter upon *dl*922-947 treatment. In 8505-c cells *dl*922-947 displaced p65 from the *CCL2* promoter (Figure 2A). BHT101-5 did not show any constitutive binding of p65 to the *CCL2* promoter (Figure 2A), likely due to an alternative regulation of *CCL2* gene expression. In accordance with *IL8* expression data, Δ19K treatment reduced *CCL2* expression in both cell lines (Supplementary Figure 3B), while transfection of E1Awt and E1AΔ24 reduced *CCL2* expression in 8505-c but not in BHT101-5 cells (Figure 3A).

To further validate our results we investigated the effects of *dl*922-947 treatment on IL-8 and CCL2 production by the papillary thyroid carcinoma cell line TPC1. *dl*922-947 and Δ19K treatments reduced IL-8 and CCL2 production at mRNA and protein levels (Supplementary Figure 4A). In addition, Δ19K significantly reduceds both *IL8* and *CCL2* expression (Supplementary Figure 4B). These results correlated with p65 displacement from *IL8* and *CCL2* promoters upon *dl*922-947 treatment (Supplementary Figure 5A). Similar to the results obtained with 8505-c cells, transfection of TPC1 cells with E1Awt or E1AΔ24 was sufficient to reduce *IL8* and *CCL2* mRNA expression (Supplementary Figure 5B).

### *dl*922-947 impairs ATC-induced angiogenesis and monocyte chemotaxis *in vitro* via reduction of IL-8 and CCL2

**Figure 4:**
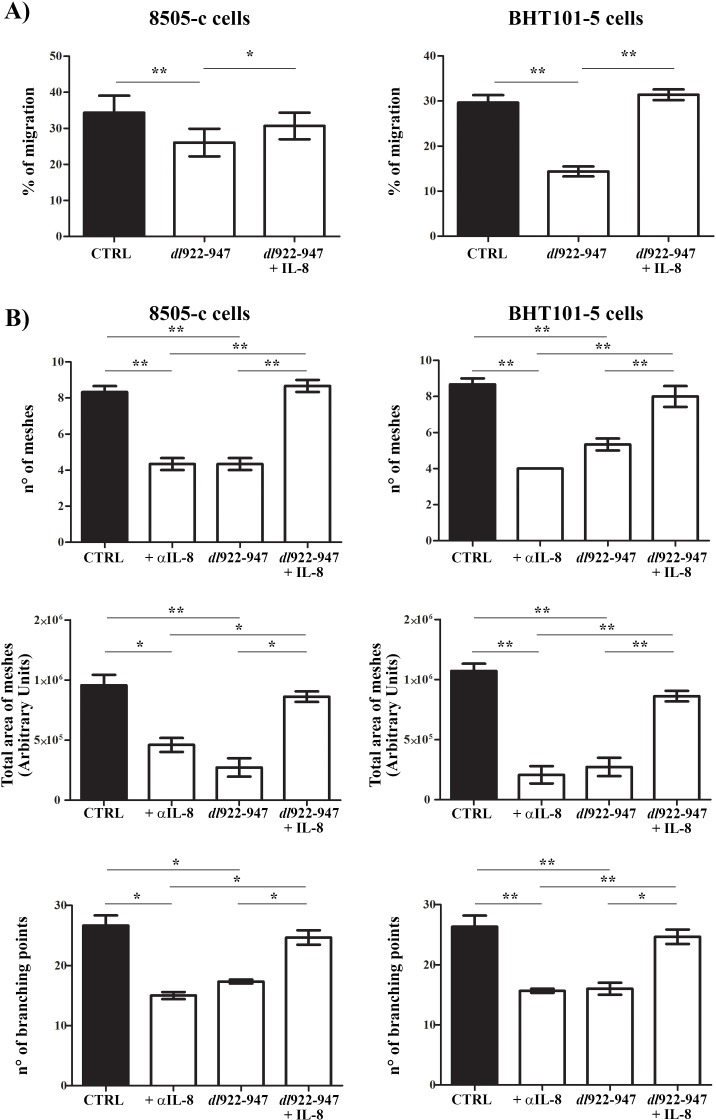
*dl*922-947 modulation of ATC cell motility and angiogenesis *in vitro* **A**. *In vitro* wound-healing assay was used to assess ATC cell motility in two dimensions. 8505-c and BHT101-5 cell monolayers were scraped in a straight line to create a “scratch”. Cells were then incubated with conditioned media (CM) obtained from 8505-c and BHT101-5 cells treated or not (CTRL) with *dl*922-947 (5 and 1 pfu/cell, respectively). Recombinant IL-8 (10 ng/mL) was also added to CM obtained from *dl*922-947-treated cells. The distance traveled by the cells was determined by measuring the wound width at time 9 hours (for 8505-c cells) and time 6 (for BHT101-5 cells) and subtracting it from the wound width at time 0. The values obtained were then expressed as % migration, setting the gap width at time 0 as 100%. One-way ANOVA and Tuckey post-test: *p<0.05; **p<0.01. **B**. HUVEC cells were incubated with CM obtained as indicated above. Tube formation was evaluated after 16 hours. Graphs display three different parameters: n° of meshes (n° of tubes), total area of meshes and n° of branching points. The results are the mean of three independent experiments ±SEM. One-way ANOVA and Tuckey post-test: *p<0.05; **p<0.01.

The remarkable reduction of IL-8 and CCL2 secretion upon *dl*922-947 treatment prompted us to investigate its impact on the outcome of ATC virotherapy. IL-8 stimulates cancer cell proliferation, survival and invasion, and angiogenesis [38]. Treatment of ATC cells with IL-8 did not affect cell cycle progression (Supplementary Figure 6A), nor it increased cell survival of *dl*922-947-treated cells (Supplementary Figure 6B). Then, we analyzed the effects of *dl*922-947 on cell motility and angiogenesis using conditioned media (CM) of untreated and *dl*922-947-treated ATC cells. *dl*922-947 CM reduced cell motility in an *in vitro* wound-healing assay (Figure 4A). The effect was reverted by the addition of recombinant IL-8 (Figure 4A). ATC-CM also had a significant pro-angiogenic activity that was impaired by the addition of an anti-IL-8 blocking antibody, as assessed by an *in vitro* angiogenesis assay (Figure 4B). Conversely, *dl*922-947-CM exhibited a reduced pro-angiogenic activity compared to CM of untreated cells (Figure 4B). The addition of recombinant IL-8 completely reverted this effect (Figure 4B).

**Figure 5:**
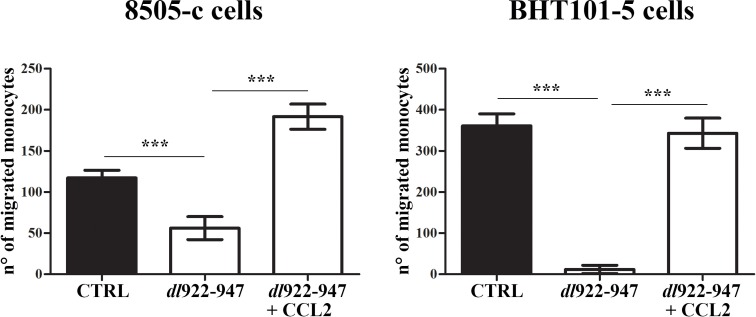
*dl*922-947 modulation of monocyte chemotaxis *in vitro* Monocytes onto the filters in the top compartment of the Boyden chamber. Conditioned media (CM) obtained from 8505-c and BHT101-5 cells treated or not (CTRL) with *dl*922-947 (5 and 1 pfu/cell, respectively) were added into the lower chamber. Recombinant CCL-2 (10 ng/mL) was also added to CM obtained from *dl*922-947-treated cells. After 2 hours filters cells that migrated through the filter were counted. The results are the mean of three independent experiments ±SEM. One-way ANOVA and Tuckey post-test: ***p<0.001.

Finally, we investigated whether ATC-CM induce monocyte chemotaxis by producing the monocyte-attracting chemokine CCL2. CM of untreated ATC cells induced monocyte chemotaxis *in vitro* (Figure 5). In accordance with *dl*922-947-mediated reduction of CCL2, *dl*922-947-CM exhibited a reduced chemotactic activity on monocytes (Figure 5). The effect was reverted by the addition of recombinant CCL2 (Figure 5).

### *dl*922-947 treatment reduces angiogenesis and TAM density in an *in vivo* model of ATC

**Figure 6:**
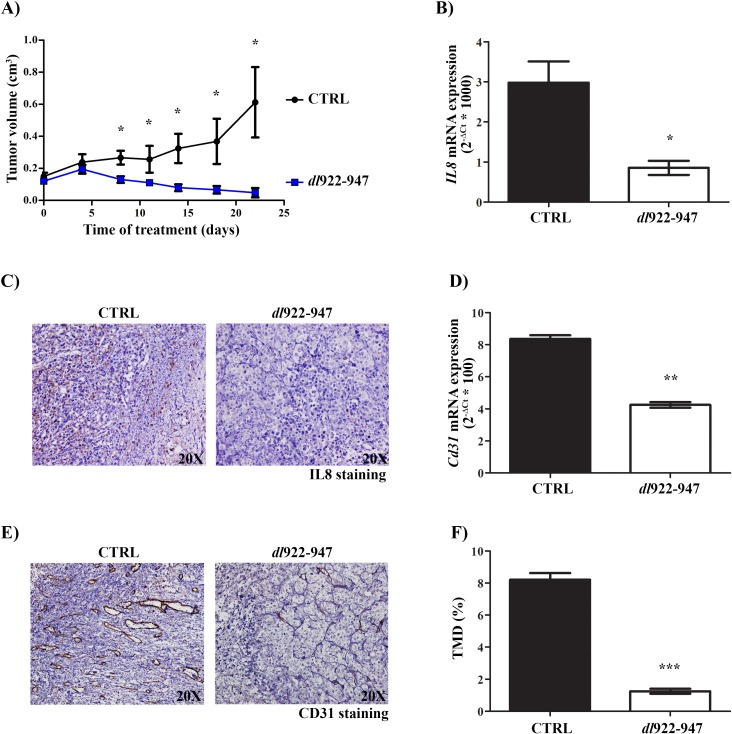
*In vivo* effects of dl922-947 on IL-8 expression and angiogenesis **A**. 6 week-old female CD1 athymic mice (15/group) were injected into the right flank with 8505-c cells (1*10^7^ cells/0.2 mL). Two weeks after tumor injection, mice were treated intratumorally with *dl*922-947 (5*10^7^ pfu) or its vehicle (CTRL) at day 0, 4, 8, 11 and 14 (day 0 = first injection of the virus). Tumor diameters were measured with calipers at day 0, 4, 8, 11, 14, 18 and 22 and tumor volume was calculated. Differences in the rate of tumor growth were assessed for each time point. Student's *t* test: *p<0.05. **B**. Real-Time PCR analysis of *IL8* mRNA levels in tumors excised at day 8 (5/group) treated as described above. Student's *t* test: *p<0.05. **C**. Representative histological analysis of IL-8 expression in tumor samples excised at day 8 treated as described above. **D**. Real-Time PCR analysis of *Cd31* mRNA levels in tumors excised at day 22 (3/group) treated as described above. Student's *t* test: **p<0.01. **E**. Representative histological analysis of CD31 expression in tumor samples excised at day 22 treated as described above. F. Quantification of tumor microvessel density (TMD) in tumor samples excised at day 22 treated as described above. TMD was determined as the percentage of CD31-positive area on total tumor area per field. Three randomly selected areas from three different tumors for each group were analyzed. Student's *t* test: ***p<0.001.

To validate our results *in vivo*, we established tumor xenografts in athymic mice using the ATC cell line 8505-c. This cell line was chosen because of its higher resistance to *dl*922-947 treatment. Mice were intratumorally injected with *dl*922-947 (5*10^7^ pfu) twice per week for a total of 3 weeks. All animals were sacrificed after 6 weeks from the beginning of treatment. *dl*922-947 treatment significantly reduced tumor volume already at 8 days and completely eradicated the tumors in 40% of mice after 2 weeks (Figure 6A and Supplementary Figure 7). These animals remained tumor-free until the end of the experiment. In previous studies performed with a different ATC cell line we did not observe a significant effect of the virus within 8 days of treatment [26, 28, 29], and a complete tumor regression rarely occurred (Portella G., personal observations). These discrepancies can be explained by taking into account the higher viral dose used in the present study.

**Figure 7:**
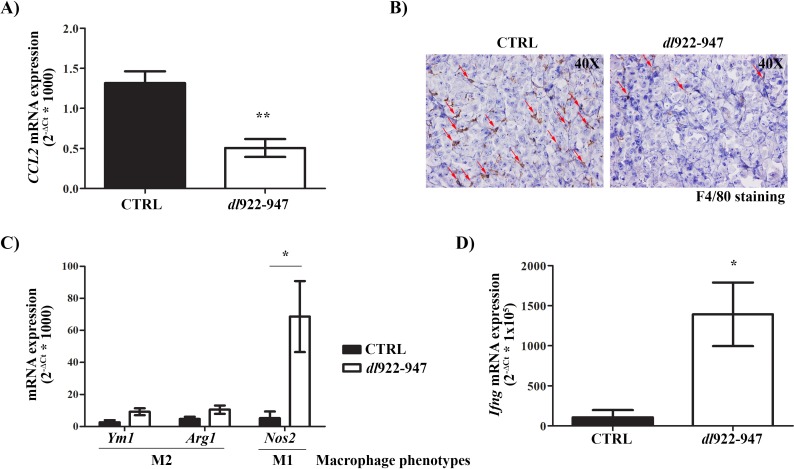
*In vivo* effects of dl922-947 on tumor macrophage density and polarization **A**. Real-Time PCR analysis of *CCL2* mRNA levels in tumors excised at day 8 (5/group) treated as described in Figure 6A. Student's *t* test: **p<0.01. **B**. Representative histological analysis of F4/80 expression in tumor samples excised at day 8 treated as described in Figure 6A. Red arrows indicate illustrative F4/80-positive cells (macrophages). **C**. Real-Time PCR analysis of mRNA levels of the M2 (*Ym1*, *Arg1*) and M1 (*Nos2*) markers in tumors excised at day 8 (5/group) treated as described in Figure 6A. Student's *t* test: *p<0.05. **D**. Real-Time PCR analysis of *Ifng* mRNA levels in tumors excised at day 8 (5/group) treated as described in Figure 6A. Student's *t* test: *p<0.05.

After 1 week of treatment, we observed decreased levels of IL-8 (Figure 6B-6C) that correlated with reduced expression of *Cd31* mRNA (an endothelial cell marker) (Figure 6D) and tumor microvessel density (TMD) (Figure 6E-6F) after 3 weeks of treatment. *CCL2* mRNA expression was also reduced after 1 week of treatment (Figure 7A). This effect was paralleled by a decrease in TAM density as shown by immunohistochemistry (Figure 7B). Interestingly, *dl*922-947 treatment also induced a switch of TAM polarization toward a pro-inflammatory M1 phenotype, as assessed by increased expression of the M1 marker *Nos2* (Figure 7C). Accordingly, we also observed increased *Ifng* mRNA levels (Figure 7D), a cytokine that induces *Nos2* expression in macrophages [52]. No significant modulation of the M2-associated genes *Ym1* and *Arg1* was detected (Figure 7C).

## DISCUSSION

Anaplastic thyroid carcinoma is one of the deadliest human malignancies, rapidly leading to trachea obstruction and death [17]. ATC is resistant to current available treatments and novel therapeutic strategies are needed. Preclinical studies have demonstrated that the oncolytic virus *dl*922-947 holds potential for the treatment of ATC. The main focus of these studies has been the direct cell killing activity of the virus and its ability to potentiate other treatments. Here, we demonstrate that *dl*922-947 treatment also affects tumor angiogenesis and macrophage density, two hallmarks of the pro-tumorigenic ATC microenvironment.

The development of a pro-tumorigenic microenvironment is a complex process, which also involves cytokine and chemokine production by cancer cells [53]. In particular, the production of IL-8 by ATC cells is involved in several aspects of tumorigenesis, namely induction of angiogenesis, epithelial-to-mesenchymal transition and stemness [35, 37]. Therapeutic strategies aimed at targeting IL-8 could represent a promising approach for ATC management. Our results demonstrate that the treatment with the oncolytic virus *dl*922-947 reduces IL-8 production by ATC cells *in vitro* and *in vivo*. In addition, we show that ATC cells produce the monocyte-attracting chemokine CCL2, which is likewise reduced by *dl*922-947 treatment. We confirmed these results in the papillary thyroid cancer cell line TPC1. The production of IL-8 and CCL2 has been demonstrated for several cancer cell types [54]. Whether the modulation of these chemokines represents a general feature of *dl*922-947 treatment or a thyroid-specific effect is unclear at present.

The expression of *IL8* and *CCL2* genes is induced by the binding of NF-κB p65 to *cis*-regulatory elements in their promoters [44-46]. We demonstrate that *dl*922-947 treatment decreases p65 binding to *IL8* promoter in both ATC cell lines and TPC1 as well. We also show that *dl*922-947 reduces p65 binding to *CCL2* promoter in 8505-c and TPC1 cells, while no constitutive p65 binding was observed in BHT101-5 cells. Several additional transcription factors (e.g. AP-1 and Sp1) [45, 46] not assessed in our study have been involved in modulating *CCL2* gene expression and likely compensate for the absence of p65 binding in BHT101-5 cells. Nevertheless, CCL2 reduction in BHT101-5 cells upon *dl*922-947 treatment suggests a broad impact of the virus on several transcription factors.

The modulation of NF-κB activity by *dl*922-947 may involve several and not mutually exclusive mechanisms. There are five NF-κB family members in mammals (RelA/p65, RelB, c-Rel, p50 and p52) that may combine in homodimers or heterodimers [55]. p65 is usually retained in the cytoplasm as a p65:p50 heterodimer due to its interaction with IκBα that masks the p65 nuclear localization signal while exposing a nuclear export signal. Phosphorylation and proteasomal degradation of IκBα induces the translocation of NF-κB dimers to the nucleus [55]. We demonstrate a direct interaction of the adenoviral protein E1A and its mutated form E1AΔ24 with p65 in 8505-c cells, a mechanism reported to inhibit the NF-κB pathway in several adenovirus-infected cell lines [48]. Transfection of both E1A and E1AΔ24 reduces *IL8* and *CCL2* mRNA expression in 8505-c cells. These results were confirmed in TPC1 cells but were not observed in BHT101-5 cells. Accordingly, we could not demonstrate the binding of E1A and E1AΔ24 to p65 in the latter cell line. *dl*922-947 treatment reduces p65 nuclear localization in BHT101-5 cells, which likely accounts for the reduced p65 binding to *IL8* promoter. Thus, it is likely that in this cell line *dl*922-947 interferes with one or more steps of p65 nuclear translocation/localization through a still uncovered mechanism. Taken together, our results indicate that *dl*922-947 interferes with the NF-κB pathway, which plays a prominent role in ATC pathogenesis [56]. Again, it will be relevant to assess whether this effect is specific for thyroid cancers or it is shared by other tumors that display an enhanced NF-κB activity (e.g. breast cancer, colorectal cancer) [57, 58].

In an effort to identify the functional significance of *dl*922-947-mediated IL-8 and CCL2 reduction, we found that *dl*922-947 treatment of ATC cells impairs IL-8-induced angiogenesis and CCL2-induced monocyte chemotaxis *in vitro*. These findings were substantiated by *in vivo* results. Indeed, *dl*922-947 treatment of tumor xenografts established in athymic mice reduces *IL8* mRNA level after 1 week and *Cd31* mRNA level and TMD after 3 weeks of treatment. These observations are in line with the anti-angiogenic effect of *dl*922-947 observed in ATC xenografts established with FRO cells [29]. Although our results strongly suggest that the anti-angiogenic activity of *dl*922-947 relies on the decreased production of IL-8 by ATC cells, we cannot exclude that the *in vivo* effects of *dl*922-947 treatment on TMD are triggered by additional mechanisms. Indeed, in unrelated tumor models, oncolytic adenoviruses exert an anti-vascular activity through different mechanisms [59]. A replication-competent oncolytic adenovirus lacking E1B suppresses the production of the pro-angiogenic factor VEGF-A in pancreatic cancer cells by disrupting the interaction between the transcription factor HIF-1α and the transcriptional co-activator p300 [60]. Our data demonstrate that *dl*922-947 does not modulate VEGF-A secretion in ATC cells. The vasculature-disrupting activity of oncolytic adenoviruses may also rely on the production of anti-angiogenic cytokines by immune cells, namely IFNγ [61].

The prominent role of TAMs in cancer progression and modulation of therapeutic responsiveness has been established in several models [62-64]. Therapeutic strategies aimed at depleting TAMs or modulating their activity are being developed and tested in clinical trials. These approaches would be suitable for treating ATC since this tumor is markedly infiltrated by TAMs [31, 32]. Treatment with wild type or E1A- or E3-deleted adenoviruses modulates macrophage infiltration in several tumor models [65]. Here we demonstrate that *dl*922-947 treatment reduces the production of the monocyte-attracting chemokine CCL2, human monocyte chemotaxis *in vitro* and TAM density *in vivo*. It is not possible to exclude that the reduced tumor vasculature in *dl*922-947-treated tumors may contribute to decrease TAM density by reducing the recruitment of peripheral blood monocytes. Thus, *dl*922-947-mediated reduction of CCL2 and IL-8 may have a synergistic effect on TAM depletion by reducing chemokine-induced monocyte recruitment and tumor vasculature.

TAM depletion in *dl*922-947-treated tumors is associated with increased expression of *Nos2*, a marker of pro-inflammatory M1 macrophages. Accordingly, we observed increased expression of *Ifng* mRNA (encoding the protein IFNγ) that may be responsible for the switch toward a macrophage M1 phenotype [52]. Several lymphocyte populations, namely NK cells, CD4 and CD8 T lymphocytes produce IFNγ. Preliminary results suggest that *dl*922-947 treatment induces intratumoral recruitment of activated NK cells (Passaro C. and Borriello F., unpublished observations). However, our results were obtained using ATC xenografts established in athymic mice. This model lacks in T cell-mediated immunity and therefore does not completely recapitulate the lymphocyte-mediated anti-tumor response elicited by virotherapy. Recently, mouse ATC cell lines have been established [66] that will allow the development of ATC allograft models in immunocompetent mice. It will be interesting to assess the effects of *dl*922-947 treatment in the context of a fully competent immune system since there is evidence that virotherapy induces a robust and long-lasting T cell-mediated anti-tumor response [7-9].

In conclusion, we demonstrate that *dl*922-947 treatment, along with its known role in inducing cell death, impacts on ATC microenvironment by modulating cancer-cell intrinsic factors and the immune response. Our results may be helpful for refining ATC virotherapy in the context of cancer immunotherapy [67].

## MATERIALS AND METHODS

### Cells, adenoviruses, recombinant cytokines and blocking antibodies

Human thyroid carcinoma cell lines 8505-c (anaplastic), BHT101-5 (anaplastic) and TPC1 (papillary) have been described and authenticated elsewhere [68]. *dl*922-947 (Δ24bpCR2E1A, ΔE3B) and the non-replicating reporter adenovirus AdGFP (ΔE1) were expanded in the human embryonic kidney cell line HEK-293, purified and stored as previously reported [69]. Δ19K was kindly provided by Dr Gunnel Halldén (Barts Cancer Institute, London, UK). Recombinant human IL-8, IFNβ and anti-IL-8 blocking antibody were purchased from PeproTech (London, UK).

### ELISA assay

1*10^4^ 8505-c, BHT101-5 or TPC1 cells were plated in 12-multiwell plates and treated as indicated. After 48 hours of treatment, cytokine concentrations were measured in cell-free supernatants using commercially available ELISA kits for IL-8 (DY208, R&D Systems, Minneapolis, MN, USA) and CCL2 (88-7399-22, eBioscience, San Diego, CA, USA). IFNβ concentration was detected by sandwich ELISA using rabbit anti-human IFNβ (500-P32B, PeproTech, London, UK) and biotinylated rabbit anti-human IFNβ (500-P32BBt, PeproTech, London, UK). When indicated, cytokine concentrations were normalized on total cellular protein content. Standard curves were generated with a Four Parametric Logistic curve fit and data were analysed using MyAssays Analysis Software Solutions (www.myassays.com).

### RNA isolation and Real-Time PCR

For *in vitro* experiments, 2*10^5^ 8505-c, BHT101-5 or TPC1 cells were plated in 60mm dishes. After 48 hours of treatment total, RNA was extracted using Trizol reagent (Invitrogen, Carlsberg, CA, USA) according to the manufacturer's instructions. For *in vivo* experiments, tumors were excised at day 8 (week 1 of treatment) and day 22 (week 3 of treatment), homogenized and total RNA was extracted using Trizol reagent (Invitrogen, Carlsberg, CA, USA) according to the manufacturer's instructions. 2 μg of total RNA were reverse-transcribed using Superscript III Reverse Transcriptase (Invitrogen, Carlsberg, CA, USA). Real-Time PCR was carried out using a CFX96 Real-Time System (Biorad, Hercules, CA, USA). Reactions were run in triplicate in three independent experiments. Specific primers were used to measure mRNA expression by Real-Time PCR. The list of primers is available as Supplementary Table 1. Expression data were normalized to the geometric mean of housekeeping gene GAPDH analyzed using the 2^−ΔCT^ or 2^−ΔΔCT^ methods [70].

### Chromatin Immunoprecipitation (ChIP) assay

2*10^6^ cells were seeded in 100mm dish and 24 hours later treated with *dl*922-947 (5, 1 and 5 pfu/cell for 8505-c, BHT101-5 and TPC1 cells, respectively). At 24 hours post-infection (hpi) cells were washed with PBS and cross-linked using 1% formaldehyde for 15 min. The cross-linking reaction was stopped by the addition of 125 mM glycine. Cells were washed twice in cold PBS, lysed in SDS lysis buffer (1% SDS, 10mM EDTA, 50mM Tris-HCl pH8.1, 1X protease inhibitor mixture) and sonicated five to nine times for 30 sec each at 18% maximum setting of the sonicator (Branson Sonifier, model 250) to achieve chromatin fragments ranging between 300 and 800 bp in size. Immunoprecipitation was carried out overnight at 4°C with 2.5 μg of the appropriate antibody: p65 (sc-372X, Santa Cruz Biotechnology, Dallas, TX, USA) or normal rabbit IgG (17-684, Millipore, Billerica, MA, USA) (as negative control). Chromatin-antibody complexes were isolated using protein G/salmon sperm DNA (Millipore, Billerica, MA, USA) and sequentially washed for 5 min each in wash buffer I (0.1% SDS, 1% Triton X-100, 2mM EDTA, 150mM NaCl, 20mM Tris-HCl, pH8.1), wash buffer II (wash buffer I with 500mM NaCl), wash buffer III (0.25M LiCl, 1% Nonidet P-40, 1% deoxycholate, 1mM EDTA, 10mM Tris-HCl, pH 8.1) and twice with TE buffer (10mM Tris-HCl, pH 7.5, 0.1mM EDTA). Input DNA (one-tenth of total lysates separated before immunoprecipitation) and immunoprecipitates were eluted by freshly prepared buffer (1% SDS, 0.1M NaHCO_3_) and overnight heated at 65°C to reverse formaldehyde cross-linking. DNA fragments were recovered by phenol/chloroform extraction and analyzed by Real-Time PCR with primers listed in Supplementary Materials. Relative fold change was assessed using the 2^−ΔΔCT^ method and normalized to input.

### Protein extraction, cellular fractionation and western blot analysis

After the indicated treatments, attached and detached cells were harvested and protein lysates were prepared as previously described [24]. 20 μg of protein lysates were probed with the following antibodies: p65 (sc-372X, 1:1000, Dallas, TX, Santa Cruz), Lamin A/C (sc-20681, 1:500, Santa Cruz, Dallas, TX, USA), α-tubulin (#T9026, 1:5000, Sigma-Aldrich, St. Louis, MO, USA), Adenovirus 2/5 E1A (sc-430, 1:500, Dallas, TX, Santa Cruz, USA). Cellular fractionation was performed using NE-PER™ Nuclear and Cytoplasmic Extraction Kit (Thermo Fisher Scientific, Waltham, MA, USA) following manufacturer's instruction.

### Cloning and transfection

The DNA sequences of wild type (E1Awt) and Δ24 form of E1A (E1AΔ24) were amplified by polymerase chain reaction (PCR) using Long expand High-Fidelity DNA Polymerase (Roche, Basel, Swiss). The viral genomic DNA from wild type adenovirus type 5 and *dl*922-947 were respectively used as templates. The following oligonucleotide primers were used:

Forward: 5′- CGATAAGCTTGTAGAGTTTTCTCCTCCGAG -3′
Reverse: 5′- CGATCTCGAGCACACACGCAATCACAGG -3′

The underlined sequences in both primer sequences correspond, respectively, to the HindIII and XhoI restriction sites, which are compatible with the multiple cloning sites on pcDNA3 expression vector. The PCR fragment and the pcDNA3 vector were then digested with the same enzymes to generate the same sticky ends. The digestion products of both the PCR fragments and the expression vector were ligated at 16°C for 16 hours in the presence of the ligation buffer and T4 DNA ligase. Ligated mixture was transformed into an appropriate bacteria strain DH5α. Transformed cells were selected on a LB plate containing 100 mg/mL ampicillin at 37°C for 16 hours. Positive transformants were inoculated into LB broth containing 100 mg/mL ampicilin for plasmids propagation. Plasmids were isolated and the presence of the inserts was determined by polymerase chain reaction (PCR) and restriction enzyme digestion. Inserts were sequenced before proceeding with transfection. pcDNA3 plasmid containing p65 coding sequence was kindly provided by Paola Ungaro (University of Naples Federico II, Naples, Italy). 8505-c, BHT101-5 and TPC1 cells were seeded in 60mm dish. When cells reached 80% of confluence, 1 μg of plasmidic DNA from p65, E1Awt and E1AΔ24 plasmids was transfected as indicated using Lipofectamine^®^ 3000 following manufacturer's instruction. (Life Technologies, Carlsbad, CA, USA).

### Co-immunoprecipitation

8505-c and BHT101-5 cells were transfected with E1Awt or E1AΔ24 alone or co-transfected with p65. 32 hours after transfection total proteins were extracted using Buffer X (50 mM Tris pH 8.8, 250 mM NaCl, 1% NP-40, 2 mM, EDTA, 2 mg/mL BSA) and 500 ng of total lysates were used for immunoprecipitation. Immunoprecipitation was carried out overnight at 4°C with 2 μg of the anti-Adenovirus 2/5 E1A antibody (sc-430, 1:500, Santa Cruz Biotechnology, Dallas, TX, USA). Protein-antibody complexes were isolated using protein A (Millipore, Billerica, MA, USA) and sequentially washed with NETN buffer (150 mM NaCl, 1 mM EDTA, 20 mM tris-HCl pH 8, 0.2 % NP-40). Buffers were supplemented with 1X proteases and phosphatases inhibitor mixture. The binding of E1Awt or E1AΔ24 to p65 was assessed by western blot using an anti-p65 antibody (sc-372X, Santa Cruz Biotechnology, Dallas, TX, USA).

### *In vitro* wound healing assay

8505-c and BHT101-5 cells were plated overnight to achieve a subconfluent cell layer in 60mm plates. Cell monolayer was scraped in a straight line to create a “scratch” with a p10 pipet tip. Debris were removed and the edge of the scratch smoothed by washing the cells once with PBS. Cells were then incubated with 3 ml of conditioned media (CM) to perform the *in vitro* scratch assay. The distance traveled by the cells was determined by measuring the wound width at time 9 or 6 hours (for 8505-c and BHT101-5 cells, respectively) and subtracting it from the wound width at time 0. The values obtained were then expressed as % of migration, setting the gap width at time 0 as 100%. CM were collected from 8505-c and BHT101-5 cells treated or not (CTRL) for 48h with *dl*922-947 and UV irradiated (seven cycles of 125J UV light) to inactivate the virus [71].

### Endothelial tube formation assay (*in vitro* angiogenesis)

1*10^4^ 8505-c or BHT101-5 cells were plated in 12-multiwell plates and treated as indicated. After 48 hours of treatment, conditioned media were collected as described above. The angiogenesis assay was performed using the Endothelial tube formation assay (*in vitro* angiogenesis) kit (Life Technologies, Carlsbad, CA, USA) following manufacturer's instruction. Three different angiogenic parameters were evaluated (number of meshes, total area of meshes and number of branching point) as previously described [72].

### *In vitro* chemotaxis assay

Peripheral blood from healthy donors was layered onto Histopaque-1077 (Sigma-Aldrich, St. Louis, MO, USA) and centrifuged at 400g for 20 min. Mononuclear cells were collected at the interface and monocytes were further purified with CD14 Microbeads (Miltenyi Biotec, Bergisch Gladbach, Germany). Chemotaxis was performed in a 48-microwell chemotaxis chamber (Costar-Nuclepore^TM^, Milan, Italy), using a 5 μm pore-size polyvinylpyrrolidone-free polycarbonate filter (Sigma-Aldrich, St. Louis, MO, USA) which divides each well into two compartments. 27 μl of CM obtained as described above were added to the lower compartment. DMEM with 0.1% BSA was used as negative control to evaluate background migration. The upper compartment was filled with 50 μl of monocyte suspension (2*10^6^ cells/ml in DMEM with 0.1% BSA). The chamber was incubated at 37°C in a 5% CO_2_ humidified atmosphere for 120 min. The filter was then removed and cells were fixed with absolute ethanol and stained with Differential Quik Stain Kit (Polysciences, Warrington, PA, USA). Cells that had not migrated were removed from the upper surface of the filter. Migrated cells were counted in nine microscopic fields per well.

### Mice and ATC xenograft model

CD-1^®^ athymic mice were obtained from Charles River (Wilmington, MA, USA) and maintained at the Department of Molecular Medicine and Medical Biotechnologies Animal Facility (University of Naples Federico II, Naples, Italy). All experiments were carried out using 6-week-old females. To initiate tumor xenografts, 8505-c cells in exponential phase were prepared at a concentration of 1*10^7^cells/ml in 0.2 ml of DMEM medium. Cell suspension was injected into the right flank of 30 animals. Tumor diameters were measured with calipers and tumor volume (V) was calculated by the formula for a rotational ellipsoid: V=A*B2/2 (A, axial diameter; B, rotational diameter). Mice weights were weekly monitored. Twenty days post-injection mice with similar tumor size were randomized into two groups (15 animals/group): untreated and treated with *dl*922–947. A high viral dose (5*10^7^ pfu) was administered twice per week by intratumoral injection to avoid any first-pass effect for a total of three weeks [26, 69, 27]. All animal experiments were conducted in accordance with accepted standards of animal care and the Italian regulations for the welfare of animals used in studies of experimental neoplasia. The study was approved by our institutional committee on animal care.

### Immunohistochemistry, tumor microvessel and TAM density measurement

Formalin fixed, paraffin embedded 4 μm sections of tumors were processed as already described [73]. Antigen retrieval was performed in 1 mM EDTA buffer pH 8 and primary antibodies were left ON at 4°C. The following antibodies were used: CD31 (Ab28364, dilution 1:50, Abcam, Cambridge, UK), F4/80 (12506, dilution 1:50, Novus, Littleton, CO, USA) and IL-8 (Ab106350, dilution 1:1000, Abcam, Cambridge, UK). To calculate the tumor microvessel density (TMD), CD31-positive area and total tumor area per field from was measured using ImageJ software. TMD was then determined as a percentage of CD31-positive area per field. Three randomly selected areas from three different tumors were analyzed.

### Statistical analysis

Statistical analysis was performed with Prism 5 (GraphPad Software). *p* values were calculated with paired *t* test or repeated measure one-way ANOVA corrected for multiple comparisons unless otherwise indicated. *p* < 0.05 was considered significant.

## SUPPLEMENTARY MATERIALS AND METHODS, FIGURES AND TABLE


