# Supplementary material for: The oncolytic virus dl922-947 reduces IL-8/CXCL8 and MCP-1/CCL2 expression and impairs angiogenesis and macrophage infiltration in anaplastic thyroid carcinoma
Source: Oncotarget. 2015 Nov 29;7(2):1500–15. doi: 10.18632/oncotarget.6430 (PMC4811476; doi:10.18632/oncotarget.6430)
Supplement: Supplementary file 1 [file oncotarget-07-1500-s001.pdf]

The oncolytic virus *dl*922-947 reduces IL-8/CXCL8 and MCP-1/CCL2 expression and impairs angiogenesis and macrophage infiltration in anaplastic thyroid carcinoma

Supplementary Materials and Methods

FACS analysis

Cells were seeded in 100 mm cell culture dish at density of $3*10^5$ cells/dish and treated as indicated. Annexin V/PI staining and cell cycle profile were analyzed as previously described [1].

References

1. Passaro C, Volpe M, Botta G, Scamardella E, Perruolo G, Gillespie D, Libertini S, Portella G. PARP inhibitor olaparib increases the oncolytic activity of dl922-947 in in vitro and in vivo model of anaplastic thyroid carcinoma. Mol Oncol. 2015; 9: 78-92.

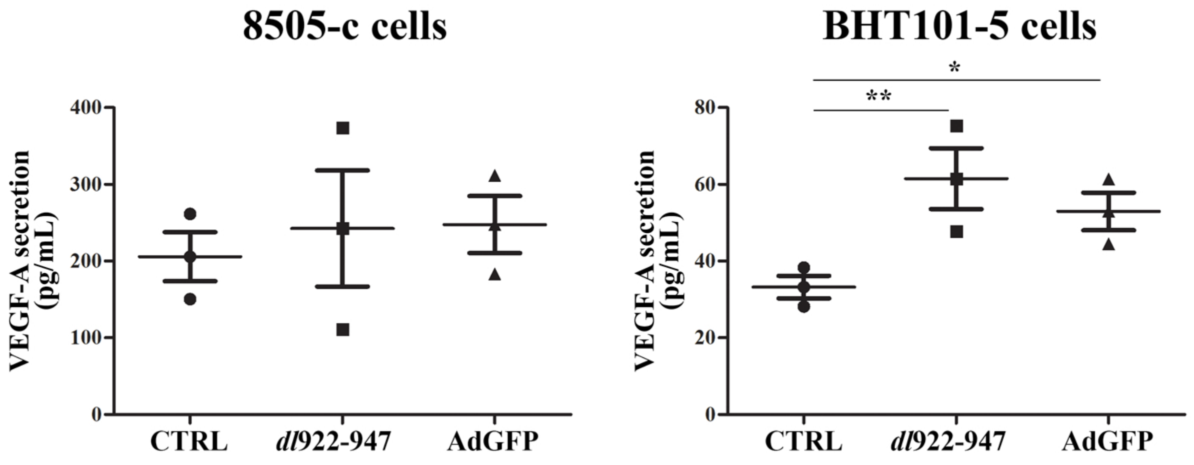

Supplementary Figure 1

A) 8505-c and BHT101-5 cells were treated with *dl*922-947 or the non-replicating adenovirus AdGFP (5 an 1 pfu/cell, respectively). 48 hours after infection VEGF-A secretion was assessed by ELISA on cell-free supernatants. The results are the mean of three independent experiments ±SEM. One-way ANOVA and Tuckey post-test: $*p<0.05$; $**p<0.01$.

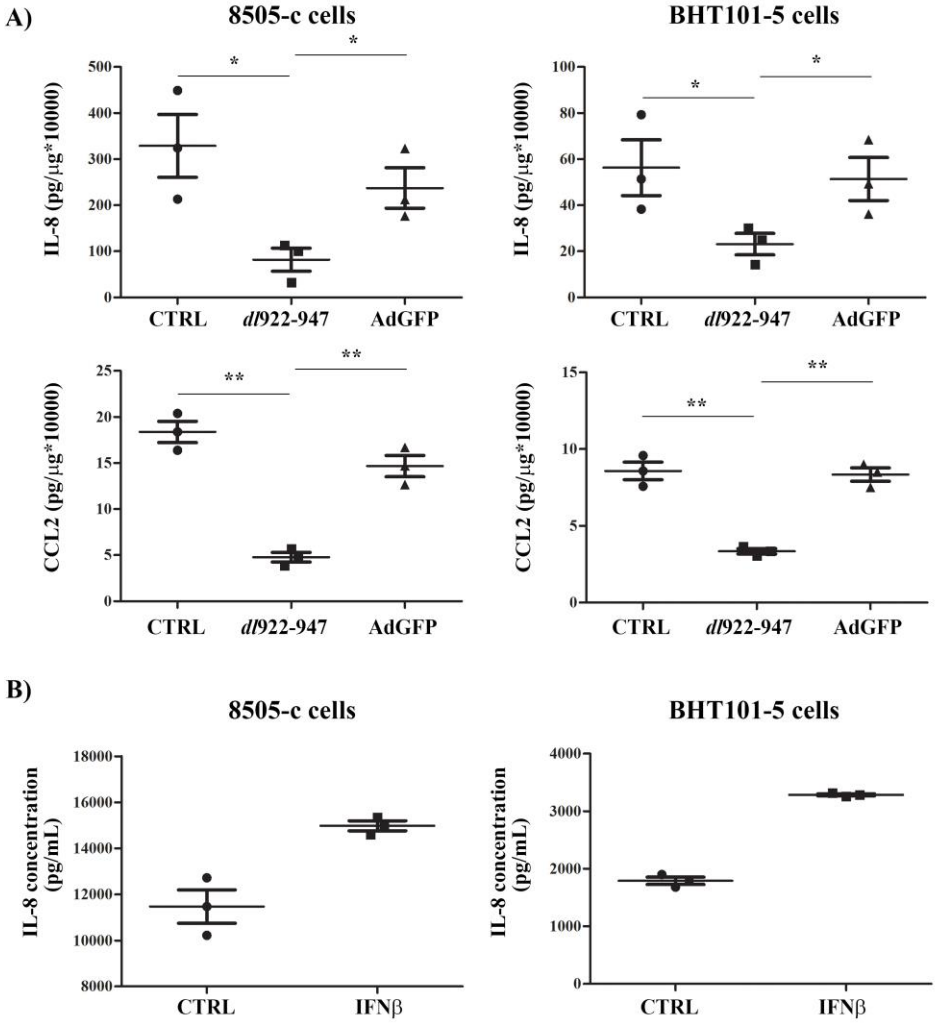

Supplementary Figure 2

A) 8505-c and BHT101-5 cells were treated with *dl*922-947 or the non-replicating adenovirus AdGFP (5 an 1 pfu/cell, respectively). 48 hours after infection IL-8 and CCL2 secretion was assessed by ELISA on cell-free supernatants. IL-8 and CCL2 levels were expressed as picograms per microgram of total cellular protein content. The results are the mean of three independent experiments ±SEM. One-way ANOVA and Tuckey post-test: *$*p<0.05$; $**p<0.01$*.

B) 8505-c and BHT101-5 cells were treated with IFNβ (10 ng/mL). After 48 hours IL-8 and CCL2 secretion was assessed by ELISA on cell-free supernatants. The results are the mean of three independent experiments ±SEM.

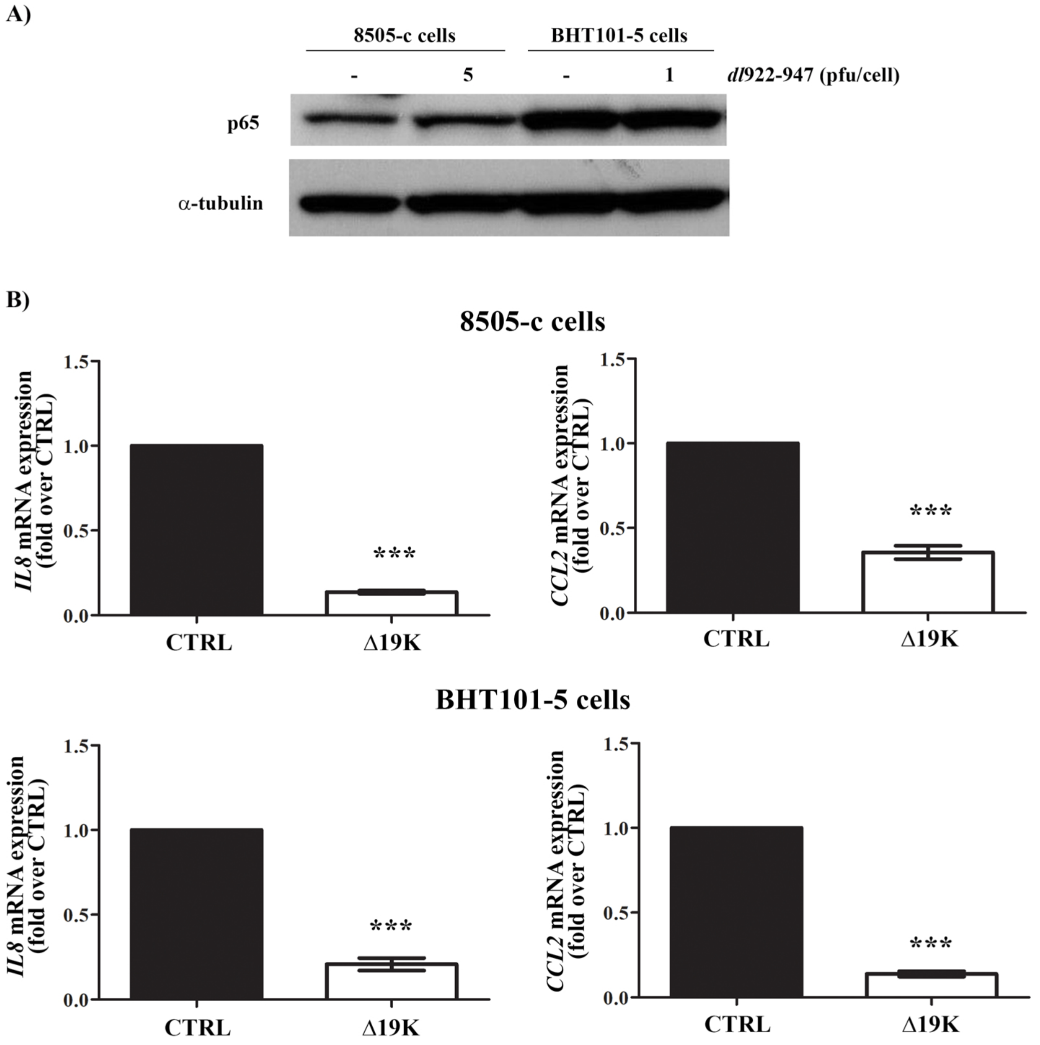

Supplementary Figure 3

A) 8505-c and BHT101-5 cells were treated for 24 hours with *dl*922-947 (5 and 1 pfu/cell, respectively). Total lysates were probed with p65 antibody. α-tubulin has been used as loading control. The blot is representative of three independent experiments.

B) 8505-c and BHT101-5 cells were treated with the viral mutant Δ19K. After 48 hours *IL8* and *CCL2* expression was assessed by Real-Time PCR. The results are the mean of three independent experiments ±SEM. One-way ANOVA and Tuckey post-test: ****p<0.001*.

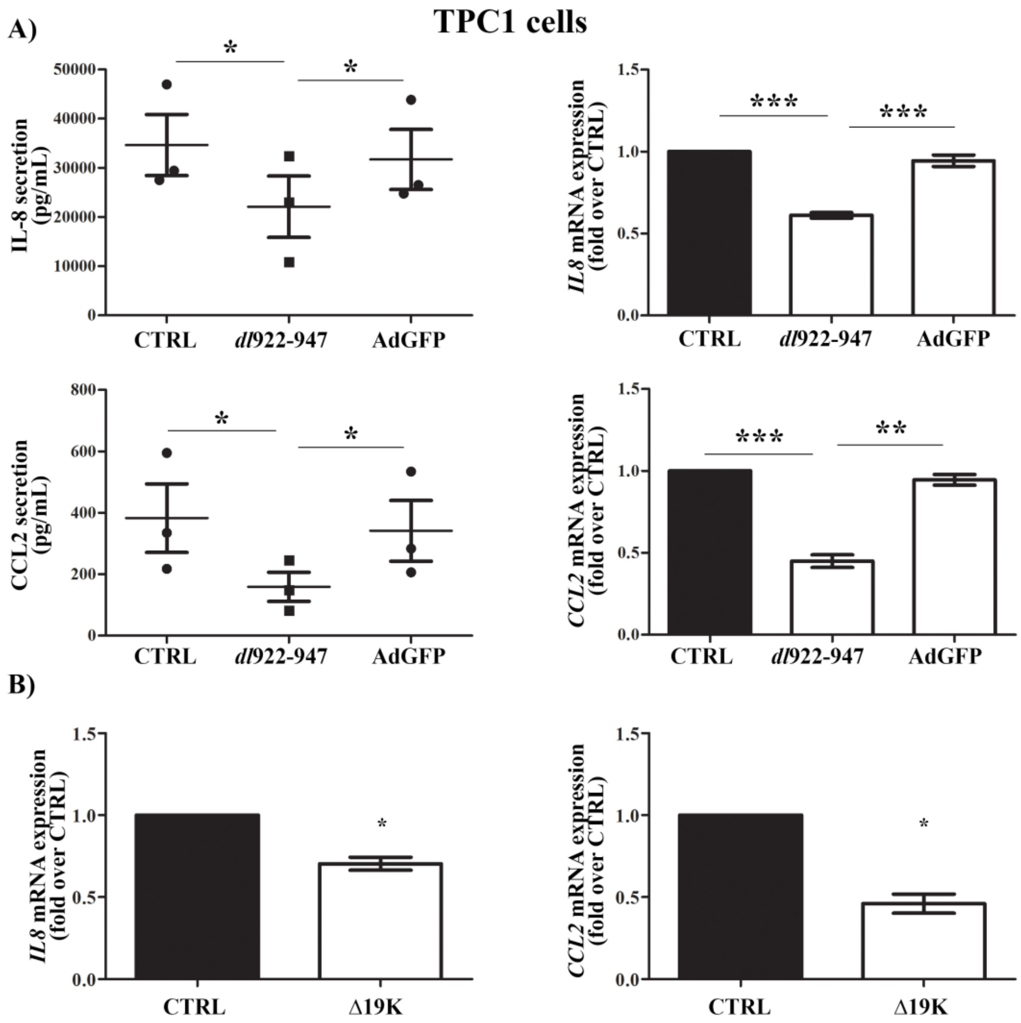

Supplementary Figure 4

A) TPC1 cells were treated with *dl*922-947 or the non-replicating adenovirus AdGFP (5 pfu/cell for both viruses). 48 hours after infection IL-8 and CCL2 secretion (left panels) and expression (right panels) were assessed by ELISA on cell-free supernatants and Real-Time PCR, respectively. The results are the mean of three independent experiments ±SEM. One-way ANOVA and Tuckey post-test: *$p<0.05$; **$p<0.01$; ***$p<0.001$.

B) TPC1 cells were treated with the viral mutant Δ19K. After 48 hours *IL8* and *CCL2* expression was assessed by Real-Time PCR. The results are the mean of three independent experiments ±SEM. One-way ANOVA and Tuckey post-test: *$p<0.05$.

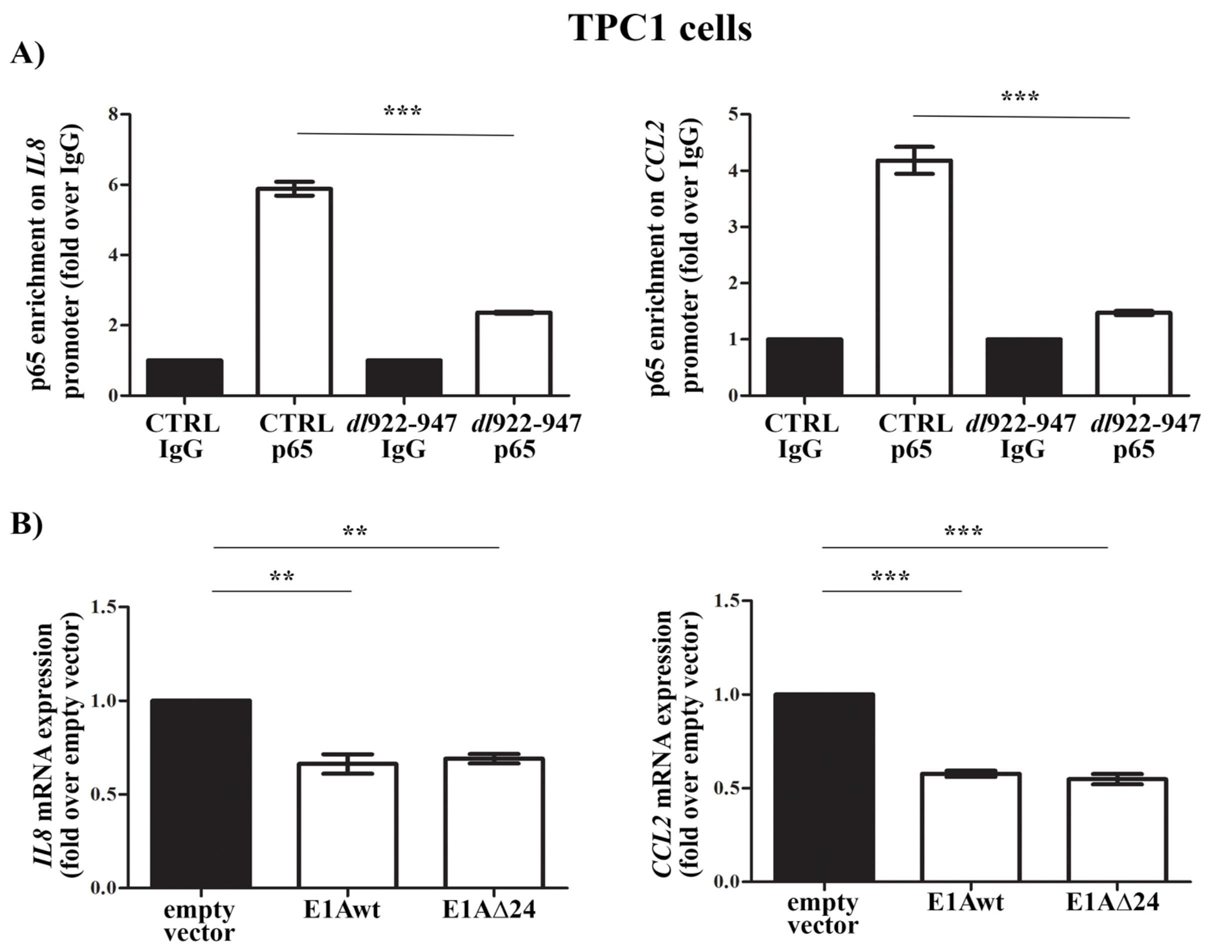

Supplementary Figure 5

A) TPC1 cells were treated with *dl*922-947 (5 pfu/mL). After 24 hours, p65 binding to *IL8* (left panel) and *CCL2* (right panel) promoters was assessed by chromatin immunoprecipitation (ChIP) assay. For each experimental condition, p65 binding is expressed as fold enrichment over the non-specific binding (IgG) control. The results are the mean of three independent experiments ±SEM. One-way ANOVA and Tuckey post-test: ***$p<0.001$.

B) TPC1 cells were transfected with wild type (E1Awt) or a mutated form (E1AΔ24) of the adenoviral protein E1A. 32 hours after transfection *IL8* and *CCL2* mRNA expression was evaluated by Real-Time PCR. The results are the mean of three independent experiments ±SEM. One-way ANOVA and Tuckey post-test: **$p<0.01$; ***$p<0.001$.

A)

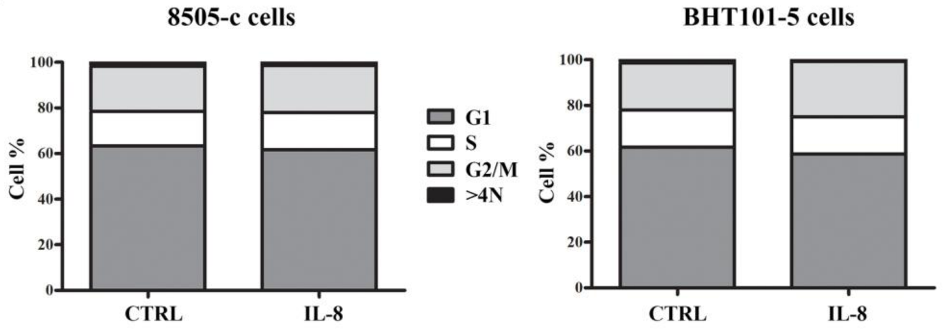

B)

8505-c cells

	Living cells	only PI +ve cells	AnnV+ve cells
CTRL	94.8 ± 2.4	0.9 ±0.2	4.3 ± 0.9
dl 922-947 (5 pfu/cell)	53.4 ± 2.8	3.3 ± 0.4	43.3 ± 3.2
IL-8 (10 ng/mL)	94.6 ± 2.0	1.8 ±0.4	3.6 ± 0.5
dl 922-947 + IL-8	49.1 ± 3.1	3.4 ± 0.8	47.3 ± 4.0
dl 922-947 + IL-8 (24hpi)	53.5 ± 2.9	4.2 ± 0.5	42.2 ± 3.4

BHT101-5 cells

	Living cells	only PI +ve cells	AnnV+ve cells
CTRL	93.3 ± 1.8	2.5 ± 0.6	4.2 ± 0.8
dl 922-947 (1 pfu/cell)	77.2 ± 1.6	5.7 ± 1.0	17.2 ± 1.1
IL-8 (10 ng/mL)	91.2 ± 1.9	4.5 ± 0.8	4.3 ± 0.5
dl 922-947 + IL-8	75.7 ± 1.5	6.5 ± 1.1	15.8 ± 1.4
dl 922-947 + IL-8 (24hpi)	75.2 ± 1.8	7.7 ± 2.3	17.1 ± 1.1

Supplementary Figure 6

A) 8505-c and BHT101-5 cells were treated or not (CTRL) with IL-8 (10 ng/mL). After 24 hours fixed cells were stained with propidium iodide and cell cycle distribution was analyzed by FACS.

The results are the mean of three independent experiments.

B) 8505-c and BHT101-5 cells were treated with *dl*922-947 (5 or 1 pfu/cell respectively) to induce cell death. Recombinant IL-8 was added at the time of infection (*dl*922-947+IL-8) or 24 hours after infection (*dl*922-947+IL-8 24 hpi). 72 hours after infection cells were collected and stained with Annexin V and propidium iodide. Annexin V posive cells (AnnV+ve) represent apoptotic cells, whereas only PI positive cells (PI+ve) are likely necrotic cell. Data in the table are the mean of three independent experiments ±SEM.

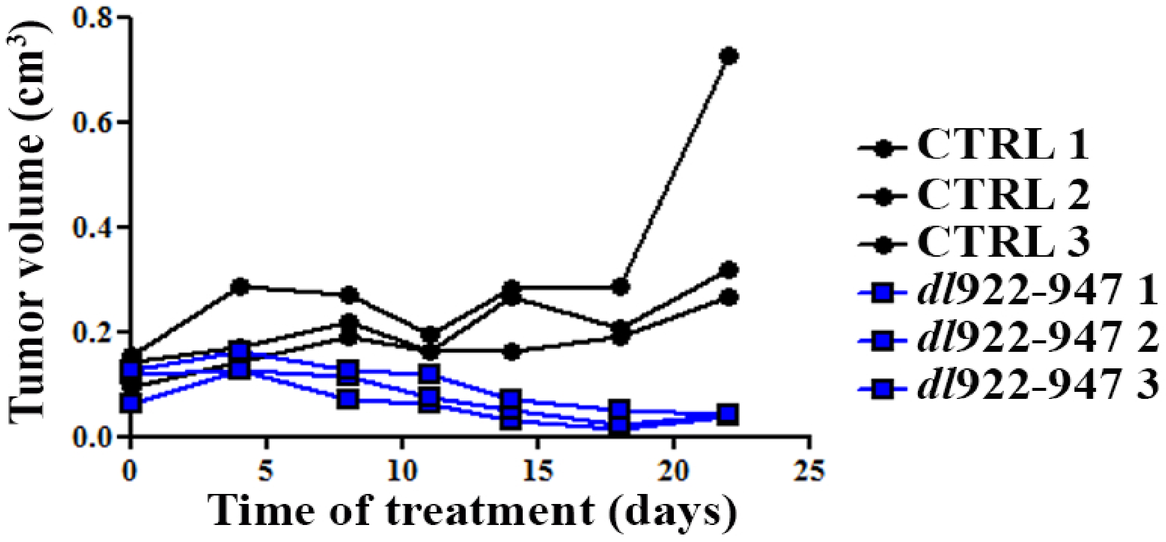

Supplementary Figure 7

6 week-old female CD1 athymic mice (15/group) were injected into the right flank with 8505-c cells ($1*10^7$ cells/0.2 mL). Two weeks after tumor injection, mice were treated intratumorally with *dl*922-947 ($5*10^7$ pfu) or its vehicle (CTRL) at day 0, 4, 8, 11 and 14 (day 0 = first injection of the virus). Tumor diameters were measured with calipers at day 0, 4, 8, 11, 14, 18 and 22 and tumor volume was calculated. The graph show the tumor growth curves for representative mice (three per group).

Supplementary Table 1

mRNA	5'→3'
human *IL8*	**Forward:** TTCCAAGCTGGCCGTGGCTC
	**Reverse:** TGTGTTGGCGCAGTGTGGTCC
human *CCL2*	**Forward:** GATCTCAGTGCAGAGGCTCG
	**Reverse:** TTTGCTTGTCCAGGTGGTCC
human *GAPDH*	**Forward:** GCATCTTCTTTTGCGTCG
	**Reverse:** GACCAAATCCGTTGACTC
mouse *Ym1*	**Forward:** AGGAAGCCCTCCTAAGGACAA
	**Reverse:** AGCCTTGGAATGTCTTTCTCCAC
mouse *Arginase 1* (*Arg1*)	**Forward:** CGGTTCTGGGAGGCCTATCT
	**Reverse:** CACCTCCTCTGCTGTCTTCC
mouse *Gapdh*	**Forward:** AAGGCCGGGGCCCACTTGAA
	**Reverse:** TGGGTGGCAGTGATGGCATGG
mouse *Nos2*	**Forward:** TCTGCAGCACTTGGATCAGG
	**Reverse:** TTCGGAAAGGGAGCAATGCCC
mouse *Interferon-γ* (*Ifng*)	**Forward:** CTTCAGCAACAGCAAGGCG
	**Reverse:** AGCGACTCCTTTTCCGCTTC
mouse *Cd31*	**Forward:** CTGCAGGCATCGGCAAA
	**Reverse:** GCATTTCGCACACCTGGAT
viral *E1A*	**Forward:** AATGGCCGCCAGTCTTTT
	**Reverse:** ACACAGGACTGTAGACAA
**HUMAN p65 BINDING SITE ON:**	**5'→3'**
IL8 promoter	**Forward:** TGGGCCATCAGTTGCAAATC
	**Reverse:** AGTGCTCCGGTGGCTTTT
CCL2 promoter	**Forward:** CCTGGAAATCCACAGGATGC
	**Reverse:** CGAGAGTGCGAGCTTCAG
